# Coronavirus Disease 2019: A Comprehensive Review of Etiology, Pathogenesis, Diagnosis, and Ongoing Clinical Trials

**DOI:** 10.7759/cureus.8076

**Published:** 2020-05-12

**Authors:** Deepika Sarvepalli

**Affiliations:** 1 Internal Medicine, Guntur Medical College, Guntur, IND

**Keywords:** sars-cov-2, covid-19, pandemic

## Abstract

Coronavirus disease 2019 (COVID-19) is an acute respiratory viral infection caused by severe acute respiratory syndrome coronavirus 2 (SARS-CoV-2). The disease outbreak started in China in late December 2019 and quickly spread to the rest of the world, resulting in a pandemic. The incidence of cases is increasing every day, affecting millions of people around the globe and resulting in a public health emergency. Furthermore, disease management has been challenging for the clinicians and other medical personnel in terms of treatment options and availability of personal protective equipment. The off-label use of drugs such as hydroxychloroquine and emergency use authorization of remdesivir can hopefully help the clinicians while treating critically ill patients. The use of convalescent serum has also shown some interim benefit until a definitive treatment and preventive options are uncovered, such as vaccines and other effective treatment regimens.

## Introduction and background

Coronavirus disease 2019 (COVID-19) is an acute respiratory viral infection related to severe acute respiratory syndrome coronavirus 2 (SARS-CoV-2). The disease outbreak started in China in late December 2019 and quickly spread to the rest of the world, resulting in a pandemic affecting 210 countries and territories worldwide so far [1]. The current mortality rate of the disease is estimated at 6.1%, with the total number of cases at 1.77 million by April 11, 2020 [1]. The incidence of new cases is increasing in devastating proportions in the United States and is declared a public health emergency [2]. This article provides a comprehensive review of the disease epidemiology, pathogenesis, diagnosis, mitigation measures, and treatment options. It also provides insights into the ongoing clinical trials aimed at limiting the disease progression.

## Review

Etiology of COVID-19

Coronaviruses are a group of viruses that infect humans, other mammals, and birds. Betacoronavirus is one of the four genera of coronaviruses and comprises some clinically important coronaviruses that can infect humans, such as severe acute respiratory syndrome coronavirus (SARS-CoV), Middle East respiratory syndrome coronavirus (MERS-CoV), and SARS-CoV-2. Coronaviruses are spherical enveloped viruses that contain single ribonucleic acid (RNA) strand (non-segmented, positive sense) and measure about 60-140 nm in diameter [3]. The outer surface contains spikes measuring 9-12 nm in length, which looks like a crown when looked under a microscope [4]. The outer surface of the virus possesses four vital structural proteins, which are spike (S), envelope (E), membrane (M), and nucleocapsid (N) proteins [5,6]. The S glycoprotein has the ability to attach to the angiotensin-converting enzyme 2 receptor (ACE-2), which helps in the fusion and eventual entry of virion particles into the host cell [5,6]. Coronaviruses are zoonotic viruses and are spread from animals to humans. There are two incidents in the past where human infections have resulted in severe disease. The first event was the 2002-2004 SARS outbreak, where humans got infected by a betacoronavirus named SARS-CoV that was usually found in bats. The epidemic started in China, affecting 8,422 people and resulting in as many as 916 deaths worldwide [7]. Later in 2012, another beta group coronavirus, MERS-CoV of bat origin, resulted in an outbreak that started in Saudi Arabia, affecting nearly 3,000 people and resulting in 858 deaths. The mortality rate of the disease was high (34%) [8].

SARS-CoV-2 pandemic timeline

In December 2019, a novel coronavirus was discovered in Wuhan city, China, which was linked to a group of pneumonia cases. Later, the virus was assumed to be transmitted by wild animals to humans, and all of the cases were associated with a seafood market [9]. On December 31, 2019, the World Health Organization (WHO) was informed by the Chinese health department about the outbreak [10]. The International Committee on Taxonomy of Viruses (ICTV) named the virus as severe acute respiratory syndrome coronavirus 2 (SARS-CoV-2) and WHO announced the name coronavirus disease 2019 (COVID-19) to refer to the disease caused by the virus [11,12]. Studies have shown that the virus is capable of spreading among humans through droplets, fomites, and direct contact. This was confirmed in an epidemiological investigation on January 20, 2020, where two patients with no travel history to Wuhan were detected with the virus in Guangdong Province, China, far from the original outbreak [13]. In January 2020, there was a rapid increase in the number of cases, which, coupled with global travel, resulted in the spread of infection around the globe. The WHO declared the outbreak as the sixth international public health emergency on January 30, 2020 [10]. By March 2020, China had reported a total of 82,160 cases of SARS-CoV-2 infection, and the mortality rate was 4%, resulting in 3,341 fatalities [14]. By the middle of February 2020, the infection spread to other countries such as Italy, Iran, United Kingdom, Spain, France, and the United States [15]. On January 20, 2020, the first case was reported in the United States in the state of Washington [16]. Since then, the infection spread to all the 50 states by March 20, 2020 [1]. By April 5, 2020, it has affected 375,000 people and resulted in almost 10,000 deaths [1]. The disease is more severe in people with underlying conditions, resulting in acute respiratory distress syndrome (ARDS) and multiorgan dysfunction [13]. As of April 9, 2020, the disease has spread to 205 countries and territories worldwide, affecting nearly 1.6 million people, and the mortality rate was estimated to be 5.95% (95,400 deaths as of April 9, 2020) [1].

Epidemiology, clinical features, and diagnosis

The initial symptoms of COVID-19 consist of fever, chills, dry cough, sore throat, nausea, headache, myalgias, which are similar to those of influenza [17]. The unique symptom reported in SARS-CoV-2 infection is the involvement of the gastrointestinal system (vomiting and diarrhea), which was not found in the case of SARS and MERS [18]. Another interesting feature of COVID-19 infection is the presence of asymptomatic carriers who can shed the virus up to 21 days without any clinical signs or symptoms. Patients with severe illness can present with shortness of breath (SOB), severe respiratory distress, and pneumonia. There is also increased reporting of new-onset anosmia and ageusia as the only presenting symptoms in some cases [19]. The average incubation period for COVID-19 is 2-14 days [19]. However, studies have reported an incubation period of as long as 19-27 days in some cases. The incubation period of COVID-19 is longer compared to that for MERS (5 days) and SARS (2-7 days). A study by Wang et al. of 138 hospitalized patients reported that the most common symptoms included fever (98.6%), dry cough (59.4%), and fatigue (69.6%). Also, in that study, the median time observed from the first symptoms to complications was five days for dyspnea, seven days for hospitalization, and eight days for ARDS [20]. These findings are helpful for clinicians who are managing at-risk populations with comorbidities where early identification of complications and timely treatment helps in patient survival. Chen et al. conducted a study of 99 hospitalized people and reported the common symptoms as fever (83%) cough (82%), and SOB (31%) [21].

The findings of the Centers for Disease Control and Prevention (CDC) report of China comprising 72,314 case records including confirmed, suspected, diagnosed, and asymptomatic cases were as follows: the overall fatality rate was 2.3% (out of 62% confirmed cases) and the mortality was higher in the elderly (15% in those aged >80 years and 8% in those aged 70-79 years) [22]. Another interesting finding of the report was that more than half of the critically ill patients had underlying comorbidities such as diabetes, chronic lung disease, cardiovascular disease, and cancer. Studies have shown that older patients with comorbidities are more susceptible to complications such as respiratory failure, multiorgan dysfunction (such as shock, acute cardiac injury, acute kidney injury), and even death in severe cases [22].

Several diagnostic tests were developed to detect the presence of COVID-19 infection, such as real-time polymerase chain reaction (PCR), reverse transcription polymerase chain (RT-PCR), reverse transcription loop-mediated isothermal amplification (RT-LAMP) assay, and enzyme-linked immunosorbent assay (ELISA) [23]. Recently in the United States, on March 28, 2020, Abbott laboratories got the FDA approval for the portable point-of-care antibody test kit that can deliver test results within 15 minutes [24]. Studies have shown that the blood cell profile of COVID-19 patients consists of decreased white blood cell (WBC) count, lymphopenia, thrombocytopenia, RNAemia, elevated aspartate aminotransferase (AST), elevated erythrocyte sedimentation rate (ESR) and C-reactive protein (CRP), and mildly elevated procalcitonin (secondary infection) levels [25]. Moreover, severe cases were associated with elevated levels of lactate dehydrogenase (LDH), alanine transaminase (ALT), creatinine, and prothrombin time [13]. Radiological findings of COVID-19 patients show a variety of changes such as ground-glass opacities, bilateral lung involvement with multiple lobular and subsegmental areas of consolidation, mottling, and pneumothorax [13]. In addition, histological findings such as hyaline membrane formation, fibromyxoid exudates, diffuse alveolar damage, and desquamation of pneumocytes were observed in critically ill patients [13,25]. The leading cause of mortality in critically ill COVID-19 happened to be due to respiratory failure from severe bilateral pneumonia [13].

Studies have shown that SARS-CoV-2 causes cytopathic damage to airway epithelial cells, which can result in the activation of severe and dysregulated immune responses, ultimately leading to ARDS [13]. Histological examination of patients with ARDS has shown hyperactive cytotoxic T-cells filled with large volumes of cytotoxic granules [13]. In addition, studies have shown that critically ill patients with COVID-19 have elevated levels of interleukin (IL) 6) due to a hyperinflammation process called cytokine storm syndrome [26]. High levels of IL-6 are responsible for shock, respiratory failure, and multiorgan failure in these patients [26]. In addition, cellular immune mechanisms are also thought to be responsible for the grave prognosis in severe disease. Damage to T lymphocytes resulting in lymphopenia and increased susceptibility to secondary infections were also observed in critically ill patients [13]. Elevation of proinflammatory cytokines along with the suppression of anti-inflammatory cytokines suggests the cellular immune response against the SARS-CoV-2 [13].

The humoral immune response has a role in the pathogenesis of COVID-19. There is a decrease in levels of immunoglobulins in COVID-19, which indicates the effects on antibody-producing B lymphocytes [21]. Though antigens of SARS-CoV-2 have shown potential for stimulating antibody production, the impact of overall lymphopenia may have caused depletion of immunoglobulins [21]. The S and N proteins on the surface of the coronavirus are thought to elicit immune responses [27]. In severely ill COVID-19 patients, the virus-specific antibodies are thought to play a role in the pathogenesis of hyperinflammation, cytokine storm, and depletion of lymphocytes through a process called antibody-dependent enhancement [28]. The blood profile of hospitalized patients showed an elevated neutrophil-lymphocyte ratio, along with a decrease in monocytes, basophils, and eosinophils [29]. In addition, a depletion of both T and B lymphocytes and natural killer (NK) cells was seen in critically ill patients, with suppression of all subsets of T cells such as helper T cells, regulatory T cells, and suppressor T cells was observed. The cytokine storm in severely ill patients results in elevation of proinflammatory cytokines in the plasma, including interferon gamma (IFN-γ), IL-1 beta, tumor necrosis factor alpha (TNF-α), granulocyte colony-stimulating factor (G-CSF), and IL-8, IL-10, IL-6, and IL-2 [29]. Therefore, detection of IL-6 can play a significant role in estimating the severity of the disease. Studies have shown that SARS-CoV-2 can result in hyperinflammation of lungs and further fibrosis by the release of IL-1β mediated by the attachment of the virions to toll-like receptors (TLR) [30]. Therefore, drugs targeting IL-1β could be potentially beneficial in severely ill patients.

Mitigation measures and clinical trials

Prevention is the mainstay of blocking the disease spread and lowering the mortality rate. Proper handwashing, maintaining at least 6 feet of social distancing while in public, and self-isolation and quarantining when suspicious of disease contraction are a few ways of preventing disease transmission. There are several ongoing studies dedicated to assessing the treatment options for COVID-19. Some of the drugs under investigation include remdesivir, chloroquine, hydroxychloroquine, tocilizumab, and convalescent serum. Wang et al. conducted in vitro studies on the SARS-CoV-2 specimen and investigated the effectiveness of antiviral drugs such as ribavirin, penciclovir, remdesivir (GS- 5734), and favipiravir (T-705), and few other medications such as nitazoxanide, nafamostat, and chloroquine in the treatment of COVID-19 [31]. The findings of their study showed that remdesivir and chloroquine had the potential of inhibiting the virus in cell cultures. Remdesivir is a nucleotide analog that exhibits antiviral properties through incorporation into the nascent viral RNA chains and further resulting in their early termination. Studies of remdesivir on the mice cell lines and nonhuman primate (NHP) cell lines have shown positive results against coronaviruses such as SARS and MERS-CoV and on Ebola virus [31]. The results of the study by Wang et al. showed that remdesivir was able to attain the required concentrations inside the monkey cell lines infected with the virus. Moreover, remdesivir was able to inhibit the infection effectively in the human liver Huh-7 cells. The second drug, chloroquine, is an immunosuppressive and anti-malarial drug traditionally used in rheumatoid arthritis and lupus. In vitro studies have shown that chloroquine has the potential to inhibit SARS-CoV-2 through mechanisms including raising the pH inside the endosomes, which prevents the fusion of the virus with the infected cell and interrupting the glycosylation of viral receptors [31]. In addition to the antiviral properties, the immunosuppressive properties of chloroquine showed a cumulative benefit in viral inhibition in cell lines [31]. With that said, in vivo studies and studies on patients infected with SARS-CoV-2 are necessary to assess the potential of these drugs in treating COVID-19 with minimal to no side effects.

In France, Gautret et al. conducted an open-label, non-randomized, small population study on hospitalized patients with COVID-19 [32]. In their study, patients were given a combination of chloroquine and azithromycin. The criteria for patient selection were age > 12 years and detection of SARS-CoV-2 antigens in the nasal swab on PCR test at admission irrespective of their clinical status [32]. Patients who were pregnant and breastfeeding and those with contraindications to chloroquine such as retinopathy, QT prolongation, and deficiency of glucose-6 phosphate dehydrogenase (G6PD) enzyme were excluded from the study. The primary endpoint of the study was a negative test result on the nasopharyngeal swab test after six days of treatment. The study enrolled 42 patients based on the above criteria, out of which 26 people were put on hydroxychloroquine and azithromycin (as needed), and the remaining 16 were put on the control group [32]. Patients were classified into three groups based on their symptoms: asymptomatic, upper respiratory tract infection (URTI) group with symptoms such as fever, body aches, rhinitis, and pharyngitis, and lower respiratory tract infections (LRTI) group with symptoms of bronchitis and pneumonia [32]. The results of the study were as follows: 100% of the patients who received both the drugs showed resolution of the viral load when compared with patients receiving hydroxychloroquine alone (57.1%) and control population (12.5%) [32]. Moreover, the effectiveness of the treatment was more significant in URTI and LRTI groups compared with asymptomatic patients.

By the end of February 2020, there were almost seven clinical trials that reviewed the effectiveness of chloroquine and hydroxychloroquine in COVID-19 people in China [33]. Liu et al. conducted in vitro studies evaluating the effect of both the drugs against SARS-CoV-2 [33]. Their study demonstrated a similar drug profile to chloroquine and hydroxychloroquine in terms of cytotoxicity, drug concentration in target cells, and tissue distribution. Previously, several clinical studies have shown that hospitalized and critically ill patients with COVID-19 exhibit excessive levels of cytokines in their plasma compared to the less symptomatic people, suggesting the role of cytokines in disease severity [33]. Hydroxychloroquine possesses good anti-inflammatory properties and is thought to be more effective in these patients. Liu et al. concluded their results by stating that hydroxychloroquine has the potential to inhibit SARS-CoV-2 infection effectively. The study also highlighted the adverse effects of the drug and its low safety index [33]. At this point, further clinical trials are necessary to assess the effectiveness of hydroxychloroquine in COVID-19.

Studies have reported that hyperactivation of humoral immune responses and release of IL-6 was observed in critically patients with COVID-19 [26]. Therefore, drugs targeting IL-6 receptors such as tocilizumab could be a potential benefit for these patients. Tocilizumab is a biological agent used in moderate-to-severe rheumatoid arthritis that acts by binding to IL-6 receptors, thereby inhibiting the effects of IL-6. In China and Italy, a non-randomized open-label study was conducted where 21 critically ill patients with COVID-19 were treated with tocilizumab [34]. All the patients were put on a standard therapy consisting of lopinavir and methylprednisolone in addition to intravenous tocilizumab 400 mg. All the patients in the study reported resolution of symptoms such as fever within 24 hours of the start of the treatment [34]. Moreover, 75% of the patients reported a significant decrease in oxygen requirement within two to five days after receiving treatment, and 90% of the patients had resolution of pneumonia radiologically as well [34]. The study did not report any side effects of the treatment. However, more evidence in terms of positive outcomes in large patient groups is necessary to substantiate the results of the study.

In late April 2020, Wang et al. published the findings of their randomized case-control study on the effectiveness of intravenous remdesivir in severely ill COVID-19 patients [35]. The study recruited 237 patients from 10 hospitals in Wuhan, China, who met the following criteria: age ≥ 18 years, lab-confirmed infection with SARS-CoV-2, onset of symptoms to enrollment ≤ 12 days, oxygen saturation ≤ 94% on room air, and chest X-ray findings positive for pneumonia. Patients who were pregnant or breastfeeding, those with complications of liver disease such as cirrhosis, elevated liver enzymes (≥5 times normal), and renal impairment, and dialysis patients were excluded from the study. Concurrent therapy with other medications such as steroids, lopinavir-ritonavir, and interferons was allowed. Clinical improvement within 28 days of starting remdesivir was the primary endpoint of the study. The study reported that patients in the remdesivir group achieved clinical improvement faster when compared with placebo in patients with symptoms for ≤10 days (hazard ratio: 1·52; 95% CI: 0·95-2·43) [35]. However, the results were not statistically significant, and the relatively small patient population might be one of the reasons. Other limitations of the study include remdesivir introduction in the late stage of the disease and insufficient data on patient recovery as remdesivir was stopped early because of adverse effects (anemia, thrombocytopenia, and elevated bilirubin) [35].

Apart from the drug trials, other notable studies include the effectiveness of passive immunization and the use of convalescent plasma/antibodies in critically ill COVID-19 patients. Antibody treatment has been successfully used in the treatment of infectious diseases in the past. A meta-analysis of the effectiveness of convalescent plasma in acute viral infections such as SARS-CoV and MERS-CoV has shown positive results in terms of decrease of viral load and case fatality rates [36]. A study in China has reported that 10 critically ill people have shown positive results when treated with antibodies from recovered people, including improved O2 saturation and decreased viral load [37]. Studies have postulated that the antibodies have the potential to neutralize the viral particles and inhibit further infection of new cells and to activate complement and phagocytosis. However, the treatment is associated with few adverse effects. In addition to ethical issues and careful donor selection, antibody treatment is associated with the risk of thrombosis (0.04-14.9%) [38]. In addition to these studies, there are some ongoing vaccine trials across the globe against the coronavirus. Finally, there are more than 600 (including withdrawn) clinical trials dedicated to the management of COVID-19. In the United States, more than 50 active studies are currently being conducted, and some of the clinical trials are shown in Table 1 [39].

**Table 1:** COVID-19: Clinical Trials in the United States COVID-19, coronavirus disease 19; SARS-CoV-2, severe acute respiratory syndrome coronavirus 2

S. No	Name of the study	Status of the trial	Drugs tested	Locations
1	Study to Evaluate the Safety and Antiviral Activity of Remdesivir (GS-5734™) in Participants With Severe Coronavirus Disease (COVID-19)	Recruiting	Remdesivir	Kaiser Permanente Los Angeles Medical Center, 3340 E. La Palma Avenue Anaheim, California, United States; Kaiser Permanente Los Angeles Medical Center, 9333 Imperial Highway Downey, California, United States; Kaiser Permanente Los Angeles Medical Center, 9961 Sierra Ave Fontana, California, United States; and 166 more
2	Study to Evaluate the Safety and Antiviral Activity of Remdesivir (GS-5734™) in Participants With Moderate Coronavirus Disease (COVID-19) Compared to Standard of Care Treatment	Recruiting	Remdesivir	Kaiser Permanente Los Angeles Medical Center, 3340 E. La Palma Avenue Anaheim, California, United States; Kaiser Permanente Los Angeles Medical Center, 9333 Imperial Highway Downey, California, United States; Kaiser Permanente Los Angeles Medical Center, 9961 Sierra Ave Fontana, California, United States; and 166 more
3	Evaluation of the Efficacy and Safety of Sarilumab in Hospitalized Patients With COVID-19	Recruiting	Sarilumab	Regeneron Study Site, New York, New York, United States
4	CD24Fc as a Non-antiviral Immunomodulator in COVID-19 Treatment	Not yet recruiting	CD24Fc	Institute of Human Virology, University of Maryland, Baltimore, Maryland, United States
5	Hydroxychloroquine Post Exposure Prophylaxis for Coronavirus Disease (COVID-19)	Not yet recruiting	Hydroxychloroquine	Columbia University Irving Medical Center, New York, New York, United States
6	The Use of PUL-042 Inhalation Solution to Reduce the Severity of COVID-19 in Adults Positive for SARS-CoV-2 Infection	Not yet recruiting	PUL-042 inhalation solution	Houston Methodist Hospital, Houston, Texas, United States
7	Nitric Oxide Gas Inhalation Therapy for Mild/Moderate COVID-19	Recruiting	Nitric oxide	Massachusetts General Hospital, Boston, Massachusetts, United States
8	Losartan for Patients With COVID-19 Requiring Hospitalization	Not yet recruiting	Losartan	Hennepin County Medical Center, Minneapolis, Minnesota, United States; M Health Fairview University of Minnesota Medical Center, Minneapolis, Minnesota, United States; University of Minnesota, Minneapolis, Minnesota, United States
9	Losartan for Patients With COVID-19 Not Requiring Hospitalization	Not yet recruiting	Losartan	Hennepin County Medical Center, Minneapolis, Minnesota, United States; M Health Fairview University of Minnesota Medical Center, Minneapolis, Minnesota, United States; University of Minnesota Minneapolis, Minnesota, United States
10	Intravenous Aviptadil for COVID-19 Associated Acute Respiratory Distress	Not yet recruiting	Aviptadil by intravenous infusion + maximal intensive care	NYU Langone Medical Center, New York, New York, United States; Rambam Health Care Campus, Haifa, Israel
11	Nitric Oxide Gas Inhalation in Severe Acute Respiratory Syndrome in COVID-19	Recruiting	Nitric oxide	Massachusetts General Hospital, Boston, Massachusetts, United States
12	Safety and Immunogenicity Study of 2019-nCoV Vaccine (mRNA-1273) to Prevent SARS-CoV-2 Infection	Recruiting	mRNA-1273	Emory Children's Center - Pediatric Infectious Diseases Decatur, Georgia, United States; Kaiser Permanente Washington Health Research Institute - Vaccines and Infectious Diseases, Seattle, Washington, United States
13	Study to Evaluate the Safety and Antiviral Activity of Remdesivir (GS-5734™) in Participants With Severe Coronavirus Disease (COVID-19)	Recruiting	Remdesivir	Hoag Memorial Hospital Presbyterian, Newport Beach, California, United States; Stanford Hospital, Stanford, California, United States; University of Chicago, Chicago, Illinois, United States; and 14 more
14	Study to Evaluate the Safety and Antiviral Activity of Remdesivir (GS-5734™) in Participants With Moderate Coronavirus Disease (COVID-19) Compared to Standard of Care Treatment	Recruiting	Remdesivir	Hoag Memorial Hospital Presbyterian, Newport Beach, California, United States; Stanford Hospital, Stanford, California, United States; University of Chicago, Chicago, Illinois, United States; and 14 more
15	Adaptive COVID-19 Treatment Trial (ACTT)	Recruiting	Remdesivir	University of Alabama at Birmingham School of Medicine - Infectious Disease, Birmingham, Alabama, United States; University of California San Diego Health - Jacobs Medical Center, La Jolla, California, United States; UCLA Medical Center - Westwood Clinic, Los Angeles, California, United States; and 34 more
16	Post-exposure Prophylaxis for SARS-Coronavirus-2	Recruiting	Hydroxychloroquine	University of Minnesota, Minneapolis, Minnesota, United States

## Conclusions

The COVID-19 outbreak has been overwhelming to the nations in terms of economy and public health aspects of curbing the disease spread and keeping the incidence of cases to the lowest level possible. Furthermore, disease management has been challenging for the clinicians and other medical personnel in terms of treatment options and the availability of personal protective equipment. The off-label use of some drugs such as hydroxychloroquine and emergency use authorization of remdesivir can hopefully help the clinicians while treating critically ill patients. The use of convalescent serum has also shown some interim benefit until a definitive treatment and preventive options are uncovered, such as vaccines and other effective treatment regimens.
